# Uterine didelphys with transverse vaginal septum in a 16-year-old female: the third case report in the medical literature

**DOI:** 10.1093/jscr/rjae532

**Published:** 2024-09-23

**Authors:** Fadi Alhalak, Mouna Baddoura, Fares Abboud, Sultaneh Haddad, Marwa Ahmed Hersi, Rand Hamed, Majd Dakhalalah Bani Hani, Yaren Jendi

**Affiliations:** University Hospital of Obstetrics and Gynecology in Damascus, Damascus, Syrian Arab Republic; Faculty of Medicine, Damascus University, Damascus, Syrian Arab Republic; Stemosis for Scientific Research, Damascus, Syrian Arab Republic; Faculty of Medicine, Damascus University, Damascus, Syrian Arab Republic; Stemosis for Scientific Research, Damascus, Syrian Arab Republic; Department of Pediatric, Children’s University Hospital, Damascus, Syrian Arab Republic; Ain Shams University, Egypt, Cairo; Faculty of Medicine, Damascus University, Damascus, Syrian Arab Republic; Yarmouk University, Irbid, Jordan; Faculty of Medicine, Tartus University, Tartus, Syrian Arab Republic

**Keywords:** Müllerian duct anomalies, amenorrhea, uterine didelphys, transverse vaginal septum

## Abstract

Müllerian duct anomalies (MDAs) are congenital disorders of the female genital tract resulting from abnormal embryological development of the Müllerian ducts. These abnormalities occur in approximately 0.5%–5.0% of the general population. The case involves a 16-year-old Middle Eastern female referred to the clinic due to primary amenorrhea and lower abdominal pain. Upon evaluation, we identified a congenital anomaly known as uterine didelphys with a transverse vaginal septum. Uterine didelphys is a type of Müllerian duct anomaly characterized by the complete duplication of the uterus, cervix, and sometimes the vagina. Our case is exceptional, as most reported instances feature a longitudinal vaginal septum with uterine didelphys, and it is rare to find both longitudinal and transverse vaginal septa. The combination of uterine didelphys with only a transverse vaginal septum is extremely rare. To the best of our knowledge, this is only the third reported case of its kind.

## Introduction

Mullerian duct anomalies (MDAs) are congenital disorders of the female genital tract that usually occur due to abnormal embryological development of the Millerian ducts. These abnormalities can include problems in development, canalization, fusion, or reabsorption, which normally occurs between 6 and 22 weeks *in utero*. Multiple sources estimate these abnormalities to be present in 0.5%–5.0% of the general population [1–3].

Septate uterus is the most common anomaly with an incidence rate of 35%, followed by bicornuate uterus at 25%, arcuate uterus 20%, unicornuate at 9.6%, and complete agenesis at 3%. A didelphys uterus was the second least common at 8.3% [2]. A didelphys uterus is formed by a complete failure of the Mullerian ducts to fuse, presenting in the appearance of two separate uterine cavities and two cervices. Moreover, a transverse vaginal septum is a rare abnormality of the female genital tract with an occurrence rate between 1/2100 and 1/7200 [2]. The most common etiology is a failure in fusion and/or channeling of the vaginal plate [2].

This report presents a unique case of a 16-year-old girl with primary amenorrhea and lower abdominal pain, which was later discovered to be due to a pelvic mass caused by a rare congenital anomaly of the female reproductive system (didelphys uterus and transverse vaginal diaphragm).

## Case presentation

A 16-year-old Middle Eastern female was referred to the clinic with complaints of primary amenorrhea and lower abdominal pain that had been gradually developing for a year. There was no history of chronic diseases, surgeries, medications, or allergies.

General examination showed a 140-cm tall female, with normally developing secondary female sex characteristics, tanner 3 breast size, and tanner 3 pubic hair. On clinical examination of the perineum, the labia, clitoris, and mons pubis were normal, and the hymen was normal without swelling or tenderness. Her laboratory findings and urinalysis were normal. A pelvic ultrasound revealed a pelvic mass measuring (7 × 7 cm) with a homogeneous, turbid fluid content.

**Figure 1:**
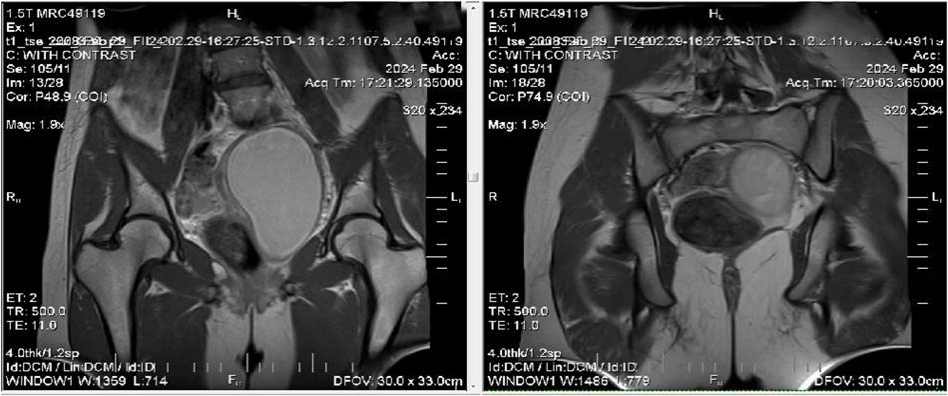
MRI imaging showing a big pelvic cystic hematoma measuring (7 cm × 12 cm × 6.7 cm) in the left paracentral position that extends close to the vaginal opening to the level of the right adnexa, encircled by a regular, thick wall that did not luminesce, and a relatively small myometrium with a regular endometrium.

**Figure 2:**
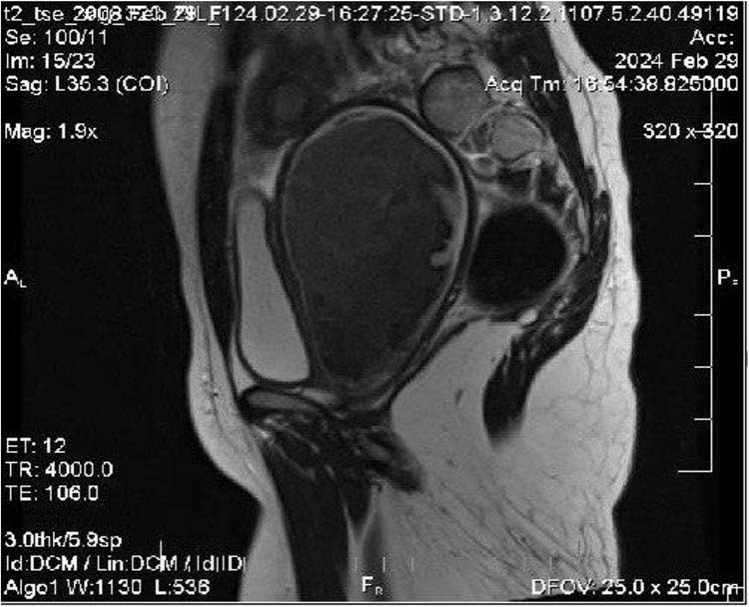
MRI imaging shows the level of vaginal obstruction.

We ordered an MRI with contrast injection to assess the ultrasound findings, which showed a big pelvic cystic hematoma measuring (7 × 12 × 6.7 cm) in the left paracentral position that extends close to the vaginal opening to the level of the right adnexa, encircled by a regular, thick wall that did not luminesce, and a relatively small myometrium with a regular endometrium (Fig. 1). It also shows the level of vaginal obstruction (Fig. 2).

**Figure 3:**
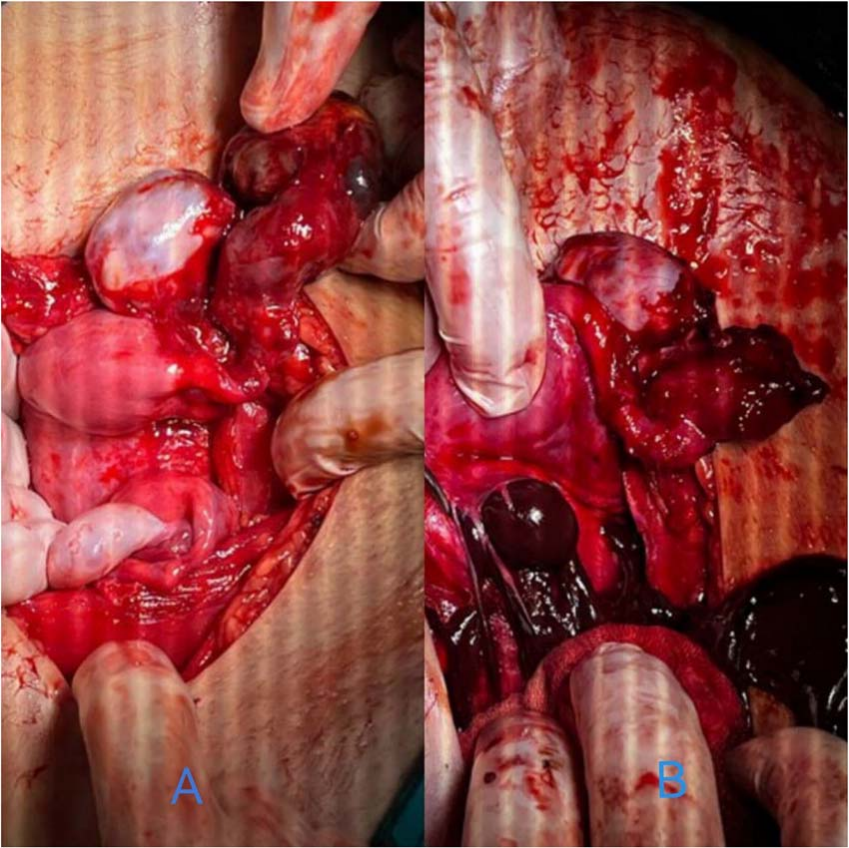
(A) A hematoma in the left fallopian tube. (B) An accumulation of blood in the vagina.

By surgically opening and reaching the abdominal cavity, two uteruses were found, along with a mass filling the pelvic cavity, measuring about (8 × 7 cm). We also saw a didelphys uterus with normal right and left ovaries, a hematoma filling the left fallopian tube, and an accumulation of blood within the vagina (hematocolpos) (Fig. 3).

**Figure 4:**
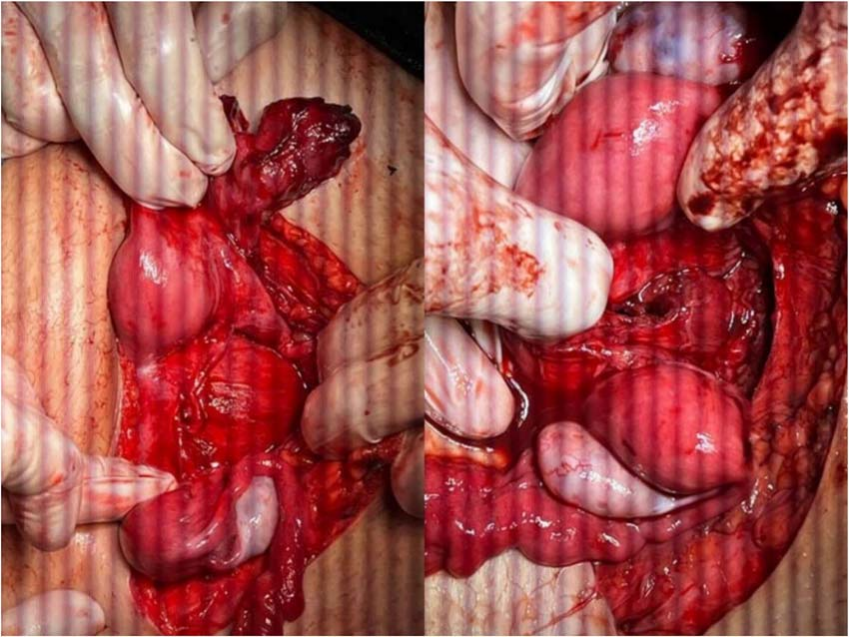
The didelphys uterus after the left fallopian tube and vaginal hematomas were drained.

The hematomas were drained (Fig. 4), and upon investigation, two cervixes were found: one for each uterus and a single vagina with a closed end, representing a transverse vaginal diaphragm.

**Figure 5:**
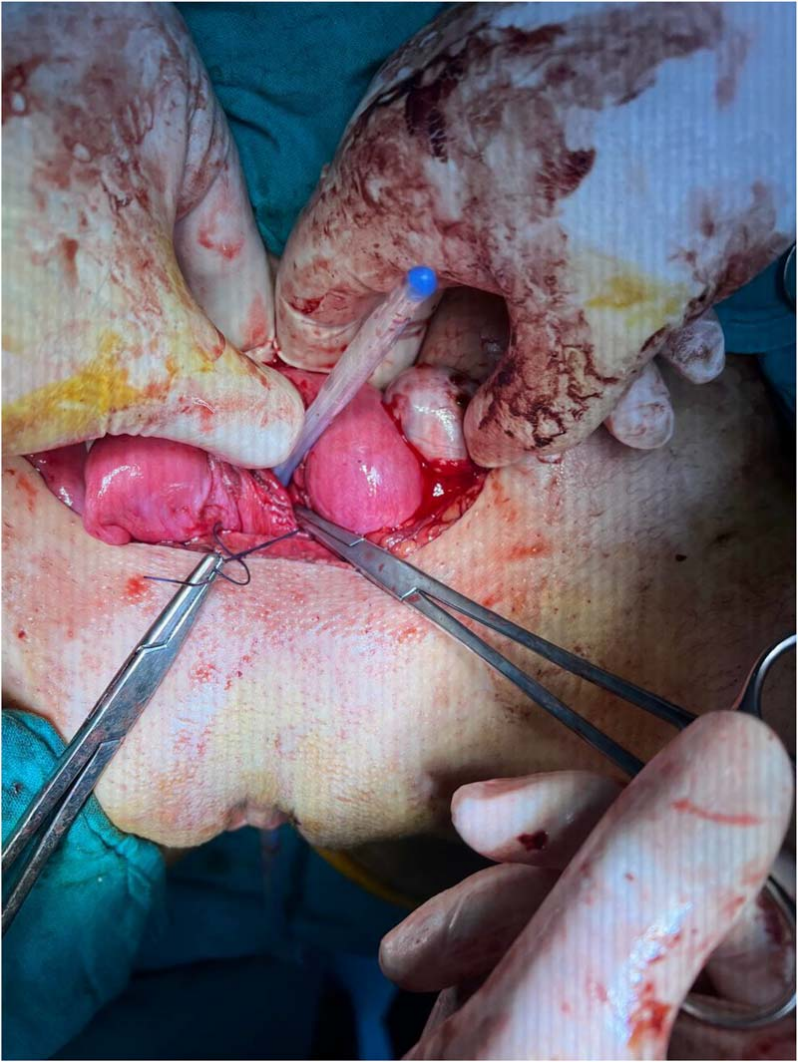
The silicon catheter placed trans-vaginally to reduce diaphragm recurrence and ensure adequate hematoma drainage.

The vaginal diaphragm was removed, the edges were sutured to reduce recurrence, and a silicone catheter was placed trans-vaginally to reduce diaphragm recurrence and ensure adequate hematoma drainage (Fig. 5). The surgical incision was sutured across the abdomen, and the abdomen was closed. The post-operation recovery was uneventful, and the patient was discharged three days later.

## Discussion

The case presents a congenital anomaly known as uterine didelphys with transverse vaginal septum. Uterine didelphys is a type of Müllerian duct anomaly, characterized by the complete duplication of the uterus, cervix, and sometimes the vagina.

The condition described in this case report is a Mullerian duct anomaly.

It includes uterus didelphys, which is rare and one of the least common uterine abnormalities. It is estimated to occur in approximately 0.1%–3% of women worldwide. However, the occurrence of a transverse vaginal septum is even rarer [4, 5].

It is believed to arise from abnormal development of the Müllerian ducts during embryogenesis, specifically during the fusion of the Müllerian ducts between the eighth and twelfth weeks of gestation. Disruptions in this process can result in various Müllerian duct anomalies, including uterine didelphys. Genetic factors may also play a role, as evidenced by familial clustering observed in some cases [4, 5].

The patient in this case presented with primary amenorrhea and lower abdominal pain, which are common symptoms associated with Müllerian duct anomalies such as uterine didelphys. Primary amenorrhea, the absence of menstruation by the age of 16 in the absence of secondary sexual characteristics, often prompts clinical investigation for underlying anatomical abnormalities. Lower abdominal pain may result from complications such as hematocolpos, as seen in this case, where blood accumulates in the vagina due to obstruction by the transverse vaginal septum. Other symptoms may include cyclic abdominal pain, dyspareunia, and difficulty with sexual intercourse [4, 5].

The diagnosis of uterine didelphys with transverse vaginal septum typically requires a combination of clinical evaluation, imaging studies, and surgical intervention. In this case, pelvic ultrasound and MRI with contrast injection were instrumental in identifying the anatomical abnormalities and guiding surgical planning. Surgical management involved excision of the transverse vaginal septum to alleviate obstruction and drainage of the hematoma. Additionally, the placement of a silicone catheter trans-vaginally aimed to prevent the recurrence of the septum and ensure adequate drainage. Post-operative recovery was uneventful, highlighting the effectiveness of the surgical intervention in resolving the patient’s symptoms [4, 5].

Carrington *et al.* in their article mentioned a patient having uterine didelphys and obstructive transverse vaginal septum in the upper one-third of the vagina [6].

Khan *et al.* reported the second case of a 16-year-old having both uterine didelphys and obstructive transverse vaginal septum [2].

Our case is unusual, as most reported instances involve a longitudinal vaginal septum with uterus didelphys, and only rarely include both longitudinal and transverse vaginal septa. The combination of uterine didelphys with only a transverse vaginal septum is exceptionally rare. To the best of our knowledge, this is only the third reported case of its kind. Unfortunately, there is no possibility in our country to perform laparoscopy and remove the septum. A hysteroscopy has not been performed because she is a virgin.

## Conclusion

The applied management of this complex case provides valuable insights into similar cases. It emphasizes the importance of a thorough clinical and radiological evaluation in young girls presenting with primary amenorrhea. Furthermore, it pushes the need for awareness and understanding of the wide range of potential causes of primary amenorrhea. It aids in the early diagnosis and appropriate management of similar cases, ultimately improving patient outcomes.
